# Metastatic prostate adenocarcinoma to the brain – a clinicopathologic analysis of 21 cases

**DOI:** 10.1186/s13000-024-01554-6

**Published:** 2024-10-01

**Authors:** Namra Ajmal, Yutao Deng, Lawrence C. Kenyon, Mark T. Curtis, Mauro Dispagna, Joseph Izes, Li Li

**Affiliations:** 1 Department of Pathology and Genomic Medicine, Thomas Jefferson University Hospital, Philadelphia, USA; 2 Department of Pathology, Cooper University Hospital, Camden, USA; 3 Department of Urology, Thomas Jefferson University Hospital, Philadelphia, USA; 4 Physician Sciences Medical Group, Norfolk General Hospital, Norfolk, VA USA

**Keywords:** Prostate adenocarcinoma, Brain metastasis, NKX 3.1, Architecture, Cytology

## Abstract

**Background:**

Brain metastasis from prostate adenocarcinoma (PCa) is rare, often leading to death within a year. Its infrequent occurrence and atypical histopathologic features contribute to lower consideration in the differential diagnosis of tumor brain metastasis. This study aims to assess the clinical characteristics and distinctive histopathologic features of metastatic PCa in the brain for timely and enhanced diagnostic accuracy.

**Design:**

A retrospective search spanning 20 years (2003–2022) was conducted on our archives and identified 21 cases diagnosed as “metastatic prostate adenocarcinoma (mPCa)” in brain biopsies and resections. All existing slides were thoroughly reviewed to evaluate the histopathology of the mPCa.

**Result:**

The mean age at presentation for brain metastasis was 70 years. Of 21 cases, 5 were dural-based lesions, 16 were true intraparenchymal metastases, including 2 sellar/suprasellar masses, 3 frontal, 3 temporal, 3 occipital, 1 cerebellum, and 4 involving multiple brain lobes. The average interval between initial diagnosis and brain metastasis was 90.75 months. Notably, brain metastasis was the initial presentation for one patient, while another patient, initially diagnosed with prognostic grade group (GG) 2 PCa in 1/12 cores, presented with isolated brain metastasis two years later. Architecturally, tumor cells were arranged in sheets or nests in most cases; however, four cases showed histologic cribriform patterns, and five displayed papillary architecture. Cytohistology varied from uniform monomorphic to highly pleomorphic cells with prominent nucleoli (8/19) and high mitotic activity. Interestingly, 1 case showed small round blue cell morphology, another had focal areas of rhabdoid and spindle cell differentiation, and 6 had cytoplasmic clearing. Almost half of the cases (47%) showed necrosis.

**Conclusion:**

mPCa to the brain can present with variable histomorphology. Therefore, consideration of mPCa in the differential diagnosis of metastatic brain lesions, even with non-suggestive imaging, is imperative in male patients with or without a history of primary disease. Accurate and prompt diagnosis is crucial, given the recent advancements in treatment that have improved survival rates.

## Background

Prostate carcinoma ranks as the second most commonly diagnosed malignancy and the fifth leading cause of death worldwide among men [1]. WHO 5th Edition for Urinary and Genital Male Tumors broadly categorizes epithelial tumors of the prostate into glandular and squamous neoplasms. Glandular neoplasms (adenocarcinoma) are broadly classified into Intra-ductal carcinoma, acinar adenocarcinoma, ductal adenocarcinoma, and treatment-related neuroendocrine prostate carcinoma (t-NEPC). Such distinction is important not only for accurate diagnosis but also for grading and prognosis purposes. Multiple rare variants have also been reported in the literature, including signet ring–like cell acinar adenocarcinoma, pleomorphic giant cell acinar adenocarcinoma, sarcomatoid carcinoma, and prostatic intraepithelial neoplasia (PIN)-like carcinoma. In addition, acinar adenocarcinoma has some special morphological patterns, including atrophic, pseudo-hyperplastic, microcystic, foamy gland, and mucinous (colloid) [2]. T-NEPC demonstrates complete or partial neuroendocrine differentiation after androgen deprivation therapy for primary prostatic tumors. Notably, 10–15% of patients can develop complete or partial neuroendocrine differentiation, which confers a worse prognosis [2].

For prostatic adenocarcinoma, bone is the most common site of metastasis, followed by lymph nodes. Later in the disease course, visceral metastasis has been described [3]. However, primary prostatic adenocarcinoma metastasizing to the brain is exceedingly rare, as seen by the paucity of reported cases, and it often leads to death within a year. For metastatic disease, understanding tumor heterogeneity is important. The primary tumor comprises diverse cancer cell clones, some exhibiting resistance to different treatment modalities. Those cancer cells can then disseminate as individual cells or clusters, proliferating and organizing at metastatic sites in accordance with their clonal histology. As a result, morphologic changes in advanced and metastatic sites might depend on the histologic properties of the clones [4].

No detailed study has been done to analyze the histomorphology of brain metastasis specifically. In this retrospective study of patients with intracranial metastasis of prostate adenocarcinoma, we discuss comprehensive histo-morphologic features of metastatic lesions with an overview of radiologic findings and a focus on recent improvements in prognosis and survival.

## Materials and methods

Sidney Kimmel Cancer Centre’s Protocol Review and Monitoring Committee and institutional review board approved and exempted this retrospective study. A record search spanning 20 years from January 2003 to December 2022 was conducted on Thomas Jefferson University Hospital pathology data archives, utilizing the Co Path Plus and Epic software. All the cases with a diagnosis of “metastatic prostate adenocarcinoma” in central nervous system biopsies and resections were included.

In our study, the time to diagnosis of brain metastasis was defined as the period in months from the date of the initial diagnosis of prostate adenocarcinoma to the date of the detection of brain metastasis. Follow-up after brain metastasis was defined as the period in months from the date of detection of CNS metastasis to the date of the last follow-up.

Patient demographics, primary tumor grade, time to brain metastasis since the initial diagnosis, and clinical/radiologic impressions were collected from available data. Subsequently, all hematoxylin and eosin-stained slides of metastatic carcinoma were thoroughly reviewed microscopically to evaluate the tumor’s histopathology. Available immunohistochemical stained slides, including but not limited to Cam 5.2, NKX 3.1, prostate-specific antigen (PSA), prostate-specific acid phosphatase (PSAP), and Prostein, were reviewed for each case.

## Results

21 cases of metastatic prostate adenocarcinoma to the central nervous system were found over the course of 20 years, and 19 had slides available for review. Of 21 cases, five were dural-based lesions, and 16 were true intraparenchymal metastasis. Of the sixteen intraparenchymal cases, two were presented as sellar/suprasellar masses with pituitary adenoma as a pre-operative differential diagnosis. The remaining fourteen cases were distributed as follows: three in the frontal lobe, three in the temporal lobe, three in the occipital lobe, and one in the cerebellum. Additionally, four cases involved multiple brain lobes. The mean age at presentation for brain metastasis was 70 years (range: 55–86 years). The average interval between initial diagnosis and brain metastasis was 90.75 months (7 years). Interestingly, the initial presentation for one patient was brain metastasis, and another patient only had grade group 2 prostatic adenocarcinoma in 1 out of 12 cores and presented with isolated brain metastasis 2 years later.

11/21 cases had primary tumor biopsy reports available to review. The prognostic grade group (GG) of the primary lesions were as follows: GG 2 (1/11), GG 3 (2/11), GG 4 (3/11), GG 5 (5/11). Ductal features were noted in the primary lesion in 2/11 cases, and a cribriform pattern was seen in 1/11 cases.

**Fig. 1:**
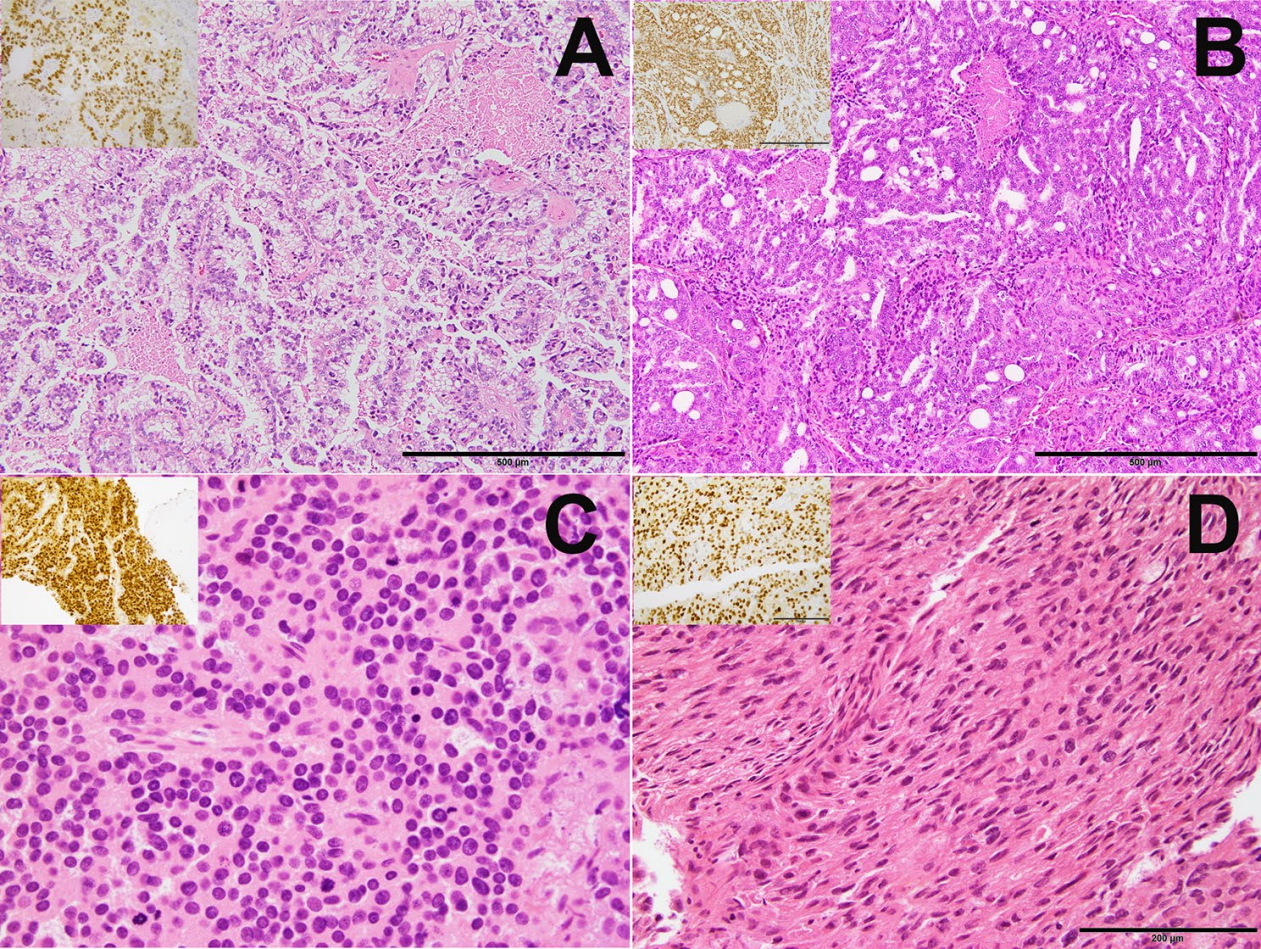
Metastatic prostate adenocarcinoma to the CNS shows different architectural patterns and cytologic morphology, including clear cell papillary architecture with focal tumor necrosis (**A**), cribriform architecture with tumor necrosis (**B**), round blue cells (**C**), and spindle cells (**D**) on Hematoxylin and eosin stain. The insets show positive nuclear staining with NKX3.1 (**A**, **B**, **C** and **D**)

Architecturally, for metastatic tumors, most cases had tumor cells arranged in sheets or nests; however, four cases showed cribriform, and five cases had papillary architecture (Fig. 1A and B). Cellular morphology varied from uniform monomorphic to highly pleomorphic cells with prominent nucleoli (8/19) with high mitotic activity. Interestingly, one case showed small round blue cell morphology, and one showed focal areas of rhabdoid and spindle cell differentiation (Fig. 1C and D). Six cases had clear cell features. Almost half of the cases (47%) showed geographic necrosis or rarely tumor comedo necrosis. Two cases also had calcifications associated with the tumor cells. No ductal features were observed in metastatic lesions, even with the primary tumor showing ductal differentiation (Fig. 1).

In our cohort of patients with brain metastasis, the other common sites for metastasis were bone (15/21), lung (4/21), and biopsy-proven lymph nodes (3/21). Rare cases of renal metastasis or mediastinal mass were also identified. Most patients had received radiation and androgen deprivation therapy for their primary lesions and radiation therapy for brain metastasis. Of 20 patients with follow-up information available, 12 died of disease ranging from within a month to 67 months (5.6 years). Unfortunately, 6 patients were lost after limited follow-up. Only three patients are alive after the brain metastases (range, 19 to 50 months) (Table 1).

## Discussion

Brain metastasis is a significant contributor to morbidity and mortality, occurring in 20% of carcinomas. Lung, breast, colorectal, or renal cell carcinoma are the most common malignant carcinomas metastasizing to the brain [5]. Among prostate adenocarcinomas, brain metastasis is exceedingly rare and seen in only 0.04 to 0.2% of cases, with most cases diagnosed at autopsy [6].

Prostatic adenocarcinoma predominantly metastasizes to bone (84%). Additional metastatic sites of prostate carcinoma include distant lymph nodes (10.6%), liver (10.2%), and thorax (9.1%). Overall, 18.4% of patients with prostate cancer have multiple metastatic sites [3]. Metastatic prostate adenocarcinoma can exhibit a range of histologic patterns, including solid, micro- or macro-acinar, clear cell, and/or tubular-papillary [7, 8]. Despite an extensive literature search, no detailed histopathologic descriptions of metastatic brain lesions were found in the studies or case reports reviewed. Most of the case reports mention at most thehistologic type of the primary prostate cancer [6, 9–12]. Twenty years ago, Tremont-Lukats conducted a comprehensive study including 131 patients diagnosed with metastatic prostatic carcinoma to the cranio-spinal region. 60% of the cases from this study were diagnosed at autopsy. Their findings indicated that 91% of patients with brain metastases had a diagnosis of prostate adenocarcinoma. However, considering the epidemiology of malignant lesions in the prostate, small cell carcinoma and cribriform subtypes were more likely than adenocarcinoma to metastasize to the brain. However, the cribriform subtype of which tumor is mentioned here is unclear [13]. All patients in their study have a prior diagnosis of prostate carcinoma at the time of brain metastasis. This is in contrast to our results, where prior diagnosis was present in 19 cases. Notably, 2 cases were first time presented with signs and symptoms of metastasis, making diagnosis more challenging. For instance, a 77-year-old healthy male with no prior medical history presented with word-finding difficulty. Imaging showed a large cystic lesion in the frontal lobe, concerning for cystic glioma or glioblastoma. Biopsies were taken, and tumor cells were strongly positive for AE1/AE3, NKX3.1, Prostein, PSA, and PSAP, confirming the diagnosis of metastatic prostate adenocarcinoma to the brain. Later, an MRI of the prostate showed a prostate imaging reporting and data system (PIRAD)5 lesion in the right posterolateral peripheral zone of the prostate, invading the right seminal vesicle and neurovascular bundle. Additional studies also revealed bilateral lung nodules and multiple bone metastasis. The patient was treated with radiotherapy to the brain and prostate and immunotherapy (Pembrolizumab and Olaparib). The patient is currently alive, 50 months after the initial diagnosis, with worsening memory loss and lower urinary tract symptoms. Another 69-year-old male presented with subdural hematoma, a soft tissue extradural mass, and multiple bone and kidney lesions. Pathology on the extradural lesion was reported as metastatic prostate carcinoma, which was strongly positive for AE1/AE3 and NKX3.1. Immunostaining for all renal and bladder markers was negative (PAX 8, p63, GATA 3), an important analysis given that the patient had radiologically identified kidney lesions. This patient unfortunately died one month following the initial diagnosis.

A recent study performed molecular characterization of intracranial prostate cancer that discussed brief histomorphology varying from solid sheets of tumor cells, dense and loose cribriform or micropapillary architecture, and/or poorly formed glands, with varying degrees of nuclear pleomorphism, mitotic activity, and necrosis [14]. This description is similar to our findings showing a vast range of morphology from bland or clear cells with acinar, cribriform, and papillary arrangement to sheets of pleomorphic cells with prominent nucleoli. Interestingly, one case with a primary diagnosis of adenocarcinoma showed features of sarcomatoid carcinoma with spindle cell and rhabdoid morphology, and another case showed round blue cell morphology. It was negative for synaptophysin and chromogranin, ruling out t-NEC. These findings could be explained by disease progression and further undifferentiation; however, tumor heterogeneity and limited sampling on biopsy missing high-grade areas could not be ruled out. NKX3.1 is considered highly specific and sensitive for metastatic prostate adenocarcinomas with a sensitivity of 98.6% and specificity of 99.7% in some studies [15]. In comparison, PSA is expressed in benign prostate tissue and in the majority of prostatic adenocarcinomas; however, it is lost in 10–20% of distant metastasis, and staining intensity decreases as compared to benign prostate tissue. The same is true for PSAP, where more than 20% metastatic prostate carcinomas lose staining with it and staining intensity is also reduced in positive staining cases [16].

The most frequent reported initial neurologic symptoms caused by intracranial metastases are confusion, headache, and memory deficits [13]. Symptoms can vary from progressive language difficulties and mixed dysphasia to hemiparesis [6]. This is consistent with our patient population, where most patients presented with non-specific neurologic symptoms, including confusion, headache, loss of consciousness, and rarely location-specific symptoms, e.g., hemianopsia or dysphagia.

On imaging, most cases were diagnosed as metastatic lesions in our study population. However, a couple of lesions in the sellar/suprasellar area were given a differential of a macroadenoma, while dural-based lesions were called meningioma. Rarely was lymphoma or glioma/glioblastoma included in the differential. Previously, a few cases were reported in literature where metastatic prostate adenocarcinoma was pre-operatively favored to be a meningioma based on PET scan [10–12]. However, it goes both ways with meningiomas being misdiagnosed as metastatic prostate carcinoma in patient with a prior prostate carcinoma history, especially on PET/CT scan due to their high ^68^Ga-PSMA uptake [9]. Interestingly, there are 8 case reports in literature so far, where tumor-to-meningioma metastasis (TMM) was seen in prostate adenocarcinoma cases [10].

Brain metastases are thought to occur via seeding of circulating tumor cells into the brain microvasculature; However, within this unique microenvironment, the penetration of systemic medical therapies is limited [5]. Molecular characterization of intracranial prostate metastasis is a new area to study for better understanding the pathogenesis and improving management options. Khani et al. recently published a detailed study showing that prostate intracranial metastasis shows a higher diversity of complex structural alterations as compared to primary tumor tissue. They also found genetic aberrations involving AR, TP53, RB1, BRCA2 genes and activation of the PI3K/AKT/PTEN pathway in multiple metastatic sites [14].

From 1944 to 1998, the median survival in untreated patients with brain metastasis was 1 month compared with 3.5 months in patients who were treated with radiotherapy. Patients who underwent stereotactic radiosurgery had a longer median survival (9 months) [13]. McCutcheon et al., in their study from (1980–1998), had a median survival of 4 months and a mean survival of 6 months if they were treated with radiation therapy [17]. It is important to note that in both these studies, all types of prostatic carcinomas were included. Our study has only metastatic prostatic adenocarcinoma, and 80% of the patients with available follow-up have died, mostly in less than 6 months, after intracranial metastasis diagnosis. One patient in our study population lived for 67 months after intracranial metastasis, the longest interval available in the literature so far.

In the context of microscopic identification of intracranial prostate adenocarcinoma metastasis, challenges arise due to the unusual anatomic site for metastasis, poor differentiation, an increased prevalence of variant morphology, a long interval from the primary lesion, and, in some cases, no documented history of a primary prostatic lesion. Detailed immunohistochemical analysis should be performed to rule out prostate adenocarcinoma in metastatic brain tumors, even in the absence of a history of primary disease or after a long interval between initial diagnosis and metastasis. With recent advances in understanding the pathogenesis and systemic therapy, there is a critical need for an accurate diagnosis so that appropriate treatment can be initiated, symptomatic relief can be provided, and long-term survival can be achieved.

**Table 1:** Demographic data and histologic features of metastatic prostatic adenocarcinoma.

Case No.	Age at time of brain metastasis	Grade group of primary tumors	Interval between initial diagnosis to CNS metastasis (months)	Imaging Findings/ Location	Histologic findings, *N*: necrosis, M: high mitotic activity	Follow up after brain metastasis (months)	Other metastatic sites.
1	70	N/A	N/A	N/A - temporal lobe lesion	Nests of pleomorphic cells with prominent nucleoli + N	DOD 1	N/A
2””	58	GG 4	< 1	Dural based lesion D/D meningioma	Nests of pleomorphic cells with prominent nucleoli + Meningioma	N/A	Retroperitoneal LNs and bones
3	86	N/A	156	N/A - temporal and occipital lobe lesion D/D metastasis	N/A	DOD < 1	N/A
4	82	N/A	237	Dural and left temporal lobe lesion D/D metastasis	Sheets and nests of clear cells + N	LTF 11	Lungs and Bones
5	69	N/A	143	Sellar and suprasellar mass D/D pituitary macroadenoma or metastasis	Sheets and nests of cells + M	DOD 7	Bones
6	55	GG 4	55	Dural and enhancing hemorrhagic anterior cranial fossa and cerebellar soft tissue mass D/D metastasis	Polygonal cells with focal clear cell morphology	LTF 6	Bones
7””	83	N/A	30	Large dural-based mass D/D metastasis	Bland cells with fibrosis and calcifications	LTF 14	Bones
8	63	N/A	241	Two hemorrhagic enhancing cerebellar masses D/D metastasis	Clear cell papillary + N	LTF 6	Lung
9””	75	N/A	23	Diffuse bony metastases with epidural mass.	Solid, acinar pattern + M	LTF 3	Bones
10	82	GG 5 (12/12)	37	Enhancing mass in the left temporoparietal region, cerebellum, and frontoparietal cortex D/D metastasis	Pleomorphic cells with Papillary and cribriform architecture + N	DOD 2	Lung and Bones
11	71	GG 5	238	Right frontal mass D/D metastasis	Papillary and cribriform architecture	DOD 10	Bones
12	57	GG 3	148	Large sellar/suprasellar lesion D/D Pituitary macroadenoma	Crushed round blue cells + N	DOD 4	Bones, LNs, and mediastinal mass
13	77	N/P	Diagnosed with brain metastasis	Cystic mass in frontal lobe D/D Cystic glioma/ glioblastoma	N/A	AWD − 50	NA
14	69	GG 5 (9/12)	57	Multiple supratentorial and infratentorial masses, large right cerebellar mass D/D metastasis	Pleomorphic and clear cells + N	DOD 3	Lungs and Bones
15* “”	69	N/P	5	Dural-based soft tissue mass, D/D meningioma or metastasis	Sheets of clear cells	DOD 1	Bones, LN’s and kidney
16	59	GG 4 (12/12)	116	Occipital hemorrhagic mass D/D metastasis	Pleomorphic cells with focal papillary architecture	DOD 5	Bones
17 “”	79	N/A	168	Dural-based frontal enhancing mass D/D meningioma or metastasis	Solid cribriform architecture + calcification + N	DOD 26	N/A
18	72	GG 5	73	Temporal lobe hemorrhagic mass with solid and cystic component	Solid cribriform and focal papillary architecture + N	AWD 19	Bones
19	77	GG 5 (10/12) IDC-P, Cribriform	19	Irregular occipital enhancing mass D/D metastasis	Nest of clear cells, focal spindle and rhabdoid differentiation + M	AWD 23	Bones
20	59	GG 2 (12/12) IDC-P	44	Bifrontal masses D/D metastasis, glioblastoma or lymphoma.	Sheets of pleomorphic cells + M + N	DOD 7	Bones and LNs
21	72	GG 3 (1/12)	24	Heterogenous occipital mass with cystic changes D/D metastasis	Pleomorphic cells	DOD 67	None

AWD: Alive with disease, D/D: Differential diagnosis, DOD: Died of disease, IDC-P: Intra ductal prostatic carcinoma present, IDC-A: Intra ductal prostatic carcinoma absent, N/A: not available, N/P: Prostate biopsy- not performed, GG: Grade Group, (*/12): number of positive cores/total number of cores biopsied, LNs: Lymph nodes, LTF: Lost to Follow up

* PC first time diagnosed in cervical lymph nodes

“” Dural-based lesions

## Data Availability

No datasets were generated or analysed during the current study.
